# Numerical Investigation of the Temperature Field Effect on the Mechanical Responses of Conventional and Cool Pavements

**DOI:** 10.3390/ma15196813

**Published:** 2022-09-30

**Authors:** Pengfei Liu, Xiangrui Kong, Cong Du, Chaohe Wang, Di Wang, Markus Oeser

**Affiliations:** 1 Institute of Highway Engineering, RWTH Aachen University, Mies-van-der-Rohe-Str. 1, 52074 Aachen, Germany; 2 Department of Civil Engineering, Aalto University, Rakentajanaukio 4, 02150 Espoo, Finland; 3 Federal Highway Research Institute (BASt), Brüderstr. 53, 51427 Bergisch Gladbach, Germany

**Keywords:** asphalt pavement, cool pavement, temperature distribution, numerical simulation, mechanical response

## Abstract

Conventional asphalt pavement has a deep surface color and large thermal inertia, which leads to the continuous absorption of solar thermal radiation and the sharp rise of surface temperature. This can easily lead to the permanent deformation of pavement, as well as aggravate the urban heat island (UHI) effect. Cool pavement with a reflective coating plays an important role in reducing pavement temperature and alleviating the UHI effect. It is of great significance to study the influence of temperature on the mechanical response of different types of pavement under vehicle loading. Therefore, this study examined the heat exchange theory between pavement and the external environment and utilized the representative climate data of a 24 h period in the summer. Two kinds of three-dimensional finite element models were established for the analysis of temperature distribution and the mechanical responses of conventional pavement and cool pavement. The results show that in this environmental condition, conventional pavement temperatures can exceed 50 °C under high temperatures in summer, which allows for the permanent deformation of pavement and further affects the service life of asphalt pavement. The temperature difference in a conventional pavement surface between 6 h (24.7 °C) and 22 h (30.2 °C) is much less than that between 22 h (30.2 °C) and 13 h (50.1 °C) in the summer. However, the difference in the vertical displacements of the pavement surface between 6 h and 22 h is much larger than that between 22 h and 13 h. One reason is that the difference in temperature distribution between the morning and night leads to changes in pavement structure stiffness, resulting in significant differences in vertical displacement. Cool pavement has a significant cooling effect, which can reduce the surface temperature of a road by more than 15 °C and reduce the vertical displacement of the pavement by approximately 11.3%, which improves the rutting resistance of the pavement. However, the use of cool pavement will not change the horizontal strain at the bottom of the asphalt base and will not improve the fatigue resistance of asphalt pavement. This research will lay the foundation for further clarifying the difference in the mechanical properties between the two types of pavements in the management and maintenance stage.

## 1. Introduction

As a typical temperature-sensitive material, the modulus of an asphalt mixture changes with its temperature. As a result, the load-carrying capacities and pavement performances of asphalt pavements are significantly influenced by temperature. Various common types of damage to asphalt pavements, such as low-temperature cracking, high-temperature rutting, and fatigue damage, are also directly or indirectly related to the temperature distribution within the pavement [1,2,3,4]. 

Compared with green vegetation, asphalt pavement has different characteristics such as a darker surface color and greater thermal inertia. Asphalt pavement constantly absorbs solar heat radiation, causing the surface temperature to rise sharply, which not only accelerates its own high-temperature rutting damage, but also exacerbates the urban heat island (UHI) effect. The use of cool pavement to mitigate the UHI effect is a popular direction in recent research. The phrase "cool pavements" was recently defined by the United States Environmental Protection Agency (USEPA) as “cool pavements include a range of established and emerging technologies that communities are exploring as part of their heat island reduction efforts. The term currently refers to paving materials that reflect more solar energy, enhance water evaporation, or have been otherwise modified to remain cooler than conventional pavements.” [5]. The cool pavement cooling principle mainly includes heat reflection, water evaporation, and road heat storage [6].

The reflective coating is a pre-treatment solution that prevents the first surface layer from absorbing heat and reflects a significant amount of energy back into the environment, thus reducing the downward conduction of heat sources in the pavement structure [7]. Zheng et al. [8] developed different reflective coating materials and studied their effects on human comfort through field tests. The results show that the use of reflective coatings can effectively improve human comfort and alleviate the UHI effect. Evaporative pavements are often designed as water-locked structures, with water absorbing heat to transition from a liquid to a gas state. This process requires the absorption of heat from the surrounding environment, which cools the pavement. Parking lots, walkways, highway shoulders, and city streets are all examples of evaporative pavements [9]. Another use of permeable pavement is for storing rainwater after high-intensity rainfall events [10]. In absorbing the heat extracted by embedded asphalt solar collectors, collected heat pavement can be used as a sustainable energy source. 

Research on the performance of asphalt pavement under a multi-physical field has been rapidly developing in recent years [11]. Wang et al. [12] studied the thermodynamics and mechanical properties of porous asphalt under high temperature and high-strength rainfall. The resistance of porous asphalt to rutting under this multi-physical field condition was evaluated. The results show that the rutting resistance of a porous asphalt mixture under rainfall conditions is lower than that under dry conditions. The rutting resistance of a porous asphalt mixture is more sensitive to temperature than to rainfall conditions. Ma et al. [13] used measured weather data to simulate temperature transfer during asphalt pavement construction. The results show that the initial temperature and layer thickness affect the overall temperature field during compaction, and wind speed and temperature mainly affect the upper-temperature field of hot mix asphalt (HMA). Zhao et al. [14] developed a three-dimensional (3D) finite element (FE) model based on transient heat transfer. The results show that the influence of temperature decreases with the increase in depth. Therefore, future subgrade temperature stress is less of a concern. Temperature differences should be considered in pavement design in regions with large temperature differences. 

In summary, a comparison of the influence of temperature on the mechanical properties of conventional pavement and cool pavement with reflective coating is seldom seen in the literature. The main objective of this paper is to study the effects of temperature on the two types of asphalt pavements using the FE method, mainly in terms of the variation of their mechanical parameters. To this end, the heat transfer model for asphalt pavement is first introduced. The development of FE models for conventional and cool pavements is then described. The results derived from the FE simulations with respect to the heat transfer within and the mechanical responses of the two types of asphalt pavements are analyzed. Finally, the conclusions and outlook are provided.

## 2. Heat Transfer Model in Asphalt Pavement

**Figure 1:**
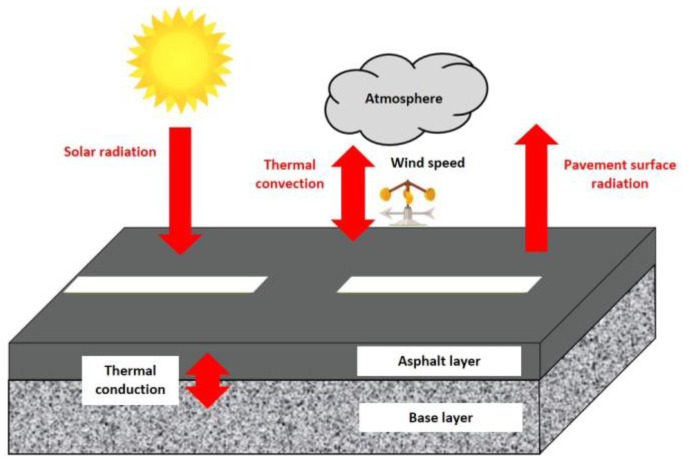
Schematic diagram of heat transfer modes for asphalt pavement.

For asphalt pavement structures, it is generally assumed that the horizontal temperature gradient is zero. Therefore, the side boundary conditions can be ignored, and the main boundaries considered are the surface boundary and the bottom boundary. The pavement surface is the main boundary of the pavement heat transfer, and the heat exchange between the pavement and the external environment is carried out mainly in three ways: solar radiation, thermal convection, and pavement surface radiation [4], as shown in Figure 1.

For solar radiation, the radiation flux incident on the surface or medium passes through three paths, namely, transmission, reflection, and absorption. According to the definition of energy conservation, the relationship between the transmissivity α, reflectivity ρ, and absorptivity τ of the radiation flux is as shown in Equation (1) [15]:

(1) α+ρ+τ=1

Asphalt pavement is a black opaque solid. For the opaque surface, the transmittance α is 0, and then ρ + τ = 1. Therefore, the lower the absorption rate is, the higher the reflectivity of the opaque surface is. The higher the reflectivity, the lower the heat loss.

According to the research of Liao et al., solar radiation can be calculated as follows [16]:

(2) $$q_{s}\left(t\right)=\left\{\begin{array}{c} 0\;\;\;\;\;\;\;\;\;\;\;\;\;\;\;\;\;\;\;\;\;\;\;\;\;\;\;\;0\leq t<12-\frac{c}{2}\\ q_{0}\cos\;m\beta (t-12)\;\;\;\;\;\;\;\;\;\;\;\;\;\;\;\;12-\frac{c}{2}\leq t\leq 12+\frac{c}{2}\\ \;0\;\;\;\;\;\;\;\;\;\;\;\;\;\;\;\;\;\;\;\;\;\;\;\;\;\;\;\;\;\;\;\;12+\frac{c}{2}\leq t<24 \end{array}\right.$$

(3) $$q_{0}=0.131{\mathrm{mQ}}_{d}$$

(4) m=12/c

where q_s_(t) is the function of heat flux of solar radiation with time (mJ/(h∙mm^2^)), q_0_ is the maximum value of q_s_, Q_d_ is the total heat flux of solar radiation per day (mJ/(mm^2^)), c is the effective duration of sunshine (h), β is a parameter (rad, 2π/24 = 0.2618), and t is the time (h).

The q_s_ values obtained by Equations (2)–(4) are not smooth and continuous, and there will be jumping points when calculating the temperature field. In accordance with the relevant principle of the Fourier series, the cosine function was expanded into the corresponding Fourier series form. When k reaches 30, it can meet the requirements of engineering accuracy [16].

(5) $$q_{s}\left(t\right)=\frac{a_{0}}{2}+\sum_{k=1}^{\infty }a_{k}\;\cos\;\frac{k\pi \left(t-12\right)}{12}\;\;\;\;\;a_{0}=\frac{2q_{0}}{m\pi }$$

(6) $$a_{k}=\left\{\begin{array}{c} \frac{q_{0}}{\pi }\;\left[\frac{1}{m+k}\;\sin\;\left(m+k\right)\;\frac{\pi }{2m}+\frac{\pi }{2m}\right]\;\;\;\;\;\;\;\;\;\;\;\;\;\;\;\;\;\;\;\;\;\;\;\;k=m\\ \frac{q_{0}}{\pi }\;\left[\frac{1}{m+k}\;\sin\;\left(m+k\right)\;\frac{\pi }{2m}+\frac{1}{m-k}\;\sin\;\left(m-k\right)\;\frac{\pi }{2m}\right]\;\;\;\;\;\;\;\;k\neq m \end{array}\right.$$

(7) $$Q_{s}=\;\tau \times {\;q}_{s}\left(t\right)$$

where Q_s_ is the effective solar radiation absorbed by the pavement mJ/(h∙m^2^), and τ is the absorptivity of solar radiation [16].

When affected by solar radiation, the air temperature will also show corresponding changes. The air temperature and wind speed are important factors affecting the heat exchange (thermal convection) between the road surface and the atmosphere. It is not accurate to use a single sine function to simulate the temperature change process during the day, and so a linear combination of two sine functions was used to simulate the temperature change process [16], as shown in Equation (8):

(8) $$T_{a}=\bar{T_{a}}+T_{m}\left[0.96\sin\omega \left(t-t_{0}\right)+0.14\sin\omega \left(t-t_{0}\right)\right],\;\omega =2\pi /24$$

where $\bar{T_{a}}$ is the average of the highest and lowest temperatures of the day, which can be expressed as [16]:

(9) $$\bar{T_{a}}=\frac{1}{2}\left(T_{a}^{\max}+T_{a}^{\min}\right)$$

where T_m_ is the magnitude of the temperature change during a day, i.e., half of the difference between the highest and lowest temperature values, which can be shown as [16]:

(10) $$T_{m}=\frac{1}{2}\left(T_{a}^{\max}-T_{a}^{\min}\right)$$

The exchange coefficient of the heat exchange, h_c_ (mJ/(h∙mm^2^ °C)), between the road surface and the atmosphere is mainly affected by the wind speed v_w_, m/s, and the relationship between them is linear [16], as shown in Equation (11):

(11) $$h_{c}=13.32v_{w}+33.84$$

The pavement surface radiation can be represented by Equation (12) [16]:

(12) $$Q_{\mathrm{ps}}=\;\varepsilon \sigma \;\left[{\left(T_{\mathrm{ps}}-T_{Z}\right)}^{4}-{\left(T_{a}-T_{Z}\right)}^{4}\right]$$

where Q_ps_ is the effective road surface radiation (mJ/(h∙mm^2^)), ε is the road surface radiation emissivity, σ = 2.04 × 10^−7^ mJ/(h∙mm^2^∙K^4^) is a Stefan-Boltzmann constant, T_ps_ and T_a_ are the pavement surface temperature and air temperature, respectively, and T_z_ is the absolute zero temperature.

Due to the influence of the atmospheric environment, the temperature fluctuation of the pavement surface is large, while the temperature fluctuation of the deeper part of the subgrade is small compared to the atmospheric environment fluctuation, which can be considered as a constant. Some researchers have considered the bottom boundary as an adiabatic boundary. However, it has been found that whether the bottom boundary condition is set to a constant temperature or is adiabatic, its effect on the temperature field of the upper layer of the asphalt pavement is negligible. 

## 3. Development of FE Model of Asphalt Pavement

Two asphalt pavement models were developed on the general-purpose FE software ABAQUS (2017, Dassault Systèmes SE, Vélizy-Villacoublay, France): one was thermal analysis, which was designed to simulate the heat transfer of asphalt pavement under the effects of solar radiation and atmospheric temperature, and the other was mechanical analysis, which had the same geometry as the first model, with the temperature field of the first model imported into this model. A time-dependent half-sine wave load was applied to observe and compare the stress and displacement distributions when considering the case of temperature variation effects and changes.

### 3.1. The FE Heat Transfer Model

**Figure 2:**
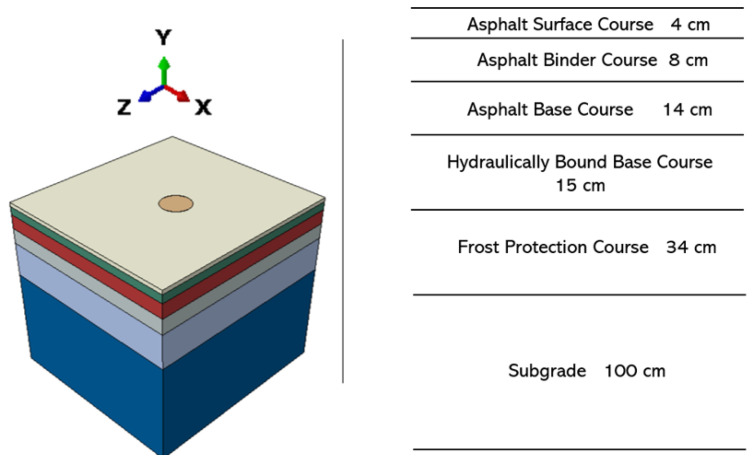
Asphalt pavement model in ABAQUS.

In this study, two FE pavement heat transfer models without (conventional pavement) and with (cool pavement or reflective pavement) reflective coatings were developed. Both models had the same pavement structure, which was divided into six layers and designed mainly according to the German design standard RStO [17]. In this standard, the width of a single-lane highway should be 3750 mm; however, a single-lane highway can be divided into two parts by the middle axis, and the left and right parts are essentially the same in structure and material properties. Therefore, the model could be simplified as an axisymmetric model, and the width of the model was set to 1875 mm. In addition, since the length of the asphalt pavement does not affect the whole temperature conduction process, the model length was selected as 2000 mm to improve the calculation efficiency of the model. To summarize, the size of the asphalt pavement model was 2000 mm × 1875 mm × 1750 mm, as shown in Figure 2. The interaction relationship between the first three layers of asphalt surface was fully connected. The vertical displacement in the last four layers of the asphalt pavement structure was set to continuous, while sliding could occur in a horizontal direction in the three interfaces.

To simulate the cooling effect of the cool pavement, Perfect Cool, a dark pavement coating with a high reflectivity (recently developed by NIPPO Corporation Co. Ltd (Tokyo, Japan)) [18] was selected. Perfect Cool aims to lower the temperature of pavement during the day by enhancing its reflectivity and minimizing the amount of heat absorbed. To decrease heat transmission, Perfect Cool combines dark, low-reflective colored pigments with high infrared-heat-reflecting pigments and small hollow ceramic particles [18]. For the FE pavement heat transfer model with a reflective coating, one additional layer with a thickness of 0.6 mm was created on top of the asphalt surface layer. The thermal parameters used by Perfect Cool are shown in Table 1.

The two modes of heat transfer—steady and transient—were set in the simulation step. The steady-state heat transfer state was set to a very small time, and the transient state simulated the temperature change process of a 24 h period.

The solar radiation acts as a surface heat flux load on the pavement models in the heat transfer model. The action surface was chosen as the upper surface of the asphalt pavement. The solar radiation fluxes defined by Equations (5)–(7) were written by the subroutine DFLUX in ABAQUS [19]. As established by the analytical Equations (8) and (11), the air temperature and wind speed will influence the simulated thermal convection. In this study, hot summer weather was considered. The specific data are shown in Table 2.

The heat exchange coefficient and air temperature were defined using the subroutine FILM in ABAQUS, according to Equations (8) and (11). In this study, the bottom boundary of the pavement structure was set to be insulated. 

The reliability of this FE heat transfer model has been validated by comparing the results from this model to the results from [16], using the same parameters. 

### 3.2. The FE Mechanical Pavement Model 

Because the reflective coating is very thin compared to the structural layers of the pavement, its influence on the mechanical response of the pavement can be ignored. Therefore, in the FE mechanical pavement modeling, only the pavement structure with six structural layers was used. The relevant mechanical parameters of the model mainly refer to RStO [17] and RDO Asphalt 09 [21], and the damping coefficient refers to the data used in the existing reference [16]. Table 3 shows the parameters of each layer of the pavement model in ABAQUS.

The temperature-dependent material properties were applied for the three asphalt layers. Due to the limited testing results, the viscoelastic parameters of the asphalt surface course and asphalt base course were derived from laboratory tests. The asphalt surface course and the asphalt base course are related to the rutting and fatigue cracking, respectively. The respective Prony series data used in ABAQUS are shown in Table 4 and Table 5. For the asphalt binder course, the temperature-dependent Young’s modulus referred to RDO Asphalt 09 [21].

**Figure 3:**
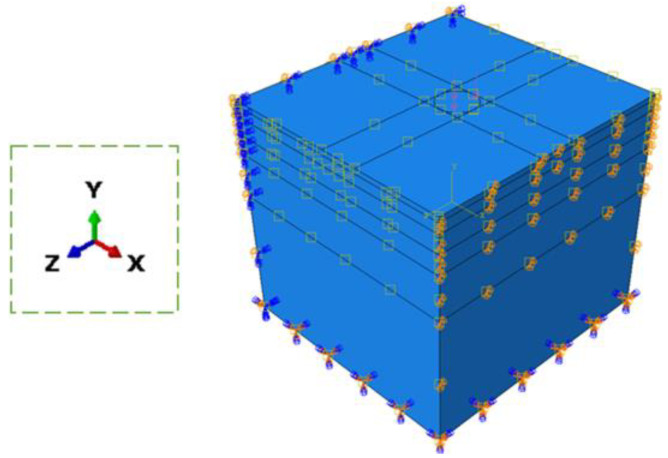
The pavement model with structural boundary conditions.

The completed heat transfer model results (temperature distribution) at specific times were imported to the mechanical model for the mechanical response of the pavement. All nodes at the bottom of the model were restricted from moving in all degrees of freedom. The four sides of the FE model were restrained from making any perpendicular movements to the side of the model. The interaction between the asphalt layers was fully coupled. The interfaces between the asphalt base course, hydraulically bound base course, frost protection course, and subgrade were assumed to be partially bound, which means that the vertical displacements of the adjacent layers were consistent, while the horizontal displacements could be different. When studying the mechanical response of the asphalt pavement model, to simplify the computational time and effect, the conventional standard load of 0.7 MPa was used. The load was applied to a circular area with a diameter of 300 mm [17]. In order to simulate the process of a tire passing over the road (45 km/h), a time-dependent half-sine wave load was created, i.e., the load was applied gradually and reached the maximum value at 0.012 s, and then the load was gradually removed until it reached zero at 0.024 s. The completed pavement model with structural boundary conditions is shown in Figure 3. The reliability of this mechanical FE model has been validated in previous investigations [22,23,24,25].

## 4. Result and Analysis

### 4.1. Results of the Responses of Conventional Pavement

#### 4.1.1. Results of the Thermal Response of Conventional Pavement

**Figure 4:**
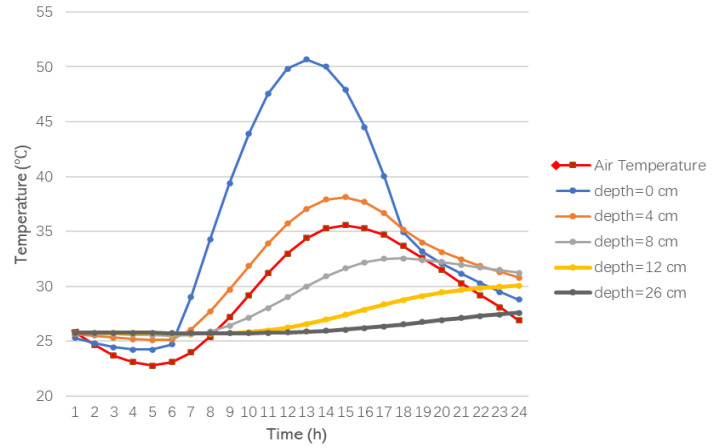
Hourly air temperature and temperature of the asphalt layers at different depths in the summer.

The analysis of the temperature changes at different depths in the model during the summer shows that the pavement heats up in summer under the effect of solar radiation. The temperature changes at different road depths within a 24 h day are shown in Figure 4.

It can be seen from Figure 4 that the maximum temperature of the road during the day reaches 50.7 °C, which is 15.6 °C higher than the air temperature at the same time. The road surface temperature is consistent with the air temperature trend, reaching the highest value at 13 h. However, with the increase in depth and the consumption of heat between the asphalt layers, the temperature variation is increasingly stable, which is approximately in the range of 25–30 °C.

**Figure 5:**
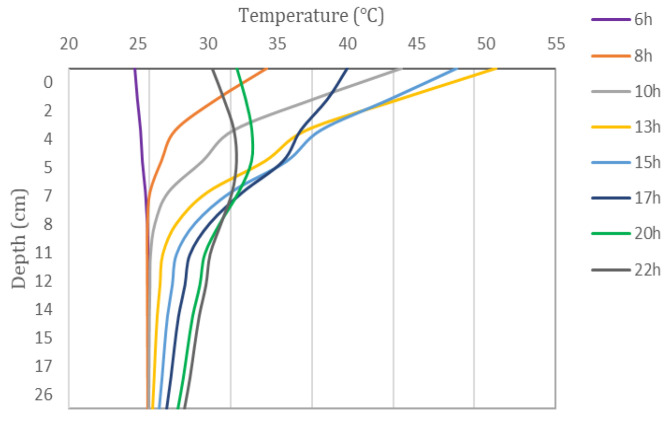
Temperature profiles of the asphalt layers along pavement depth at different moments in the summer.

The temperature curves of the asphalt layers at different times are shown in Figure 5. It can be seen that the temperature gradient varies with time and depth. With the increase in depth, the temperature is more stable with time.

The temperature difference between the top (y = 0 cm) and the bottom (y = 4 cm) of the asphalt surface course is largely positive during the day due to the absorption of solar radiation. Conversely, at night, the top temperature of the asphalt surface is higher than the air temperature, and the radiation from the pavement surface dominates the heat exchange activity. Therefore, the top temperature of the asphalt surface decreases significantly, and the temperature difference between the top and bottom of the asphalt surface is largely negative.

**Figure 6:**
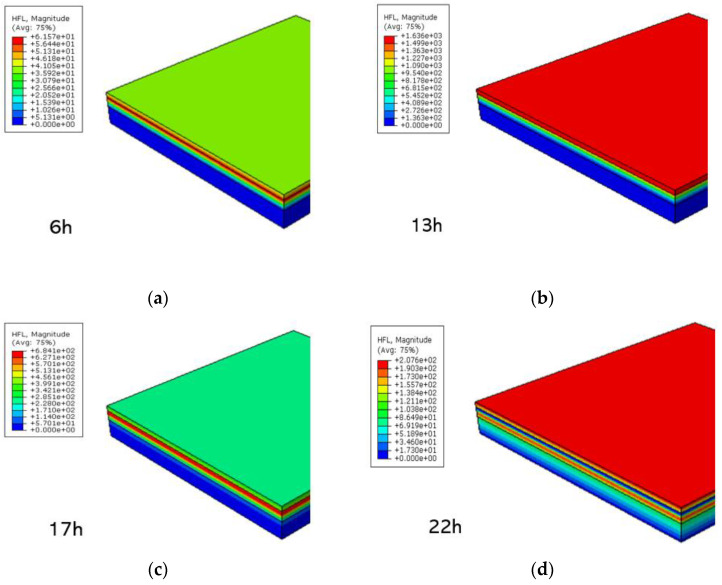
Cloud image of heat flux at different times in the summer: (**a**) 6 h, (**b**) 13 h, (**c**) 17 h, and (**d**) 22 h.

Solar radiation is applied to the surface of the road as heat flux. Heat flux refers to the heat energy passing through a unit area per unit time, and it is a directional vector. The variation of heat flux with time in the asphalt layers is shown in Figure 6. It reaches its highest at 13 h, and the variation in heat flux is consistent with the variation in temperature.

#### 4.1.2. Results of the Mechanical Response of Conventional Pavement

**Figure 7:**
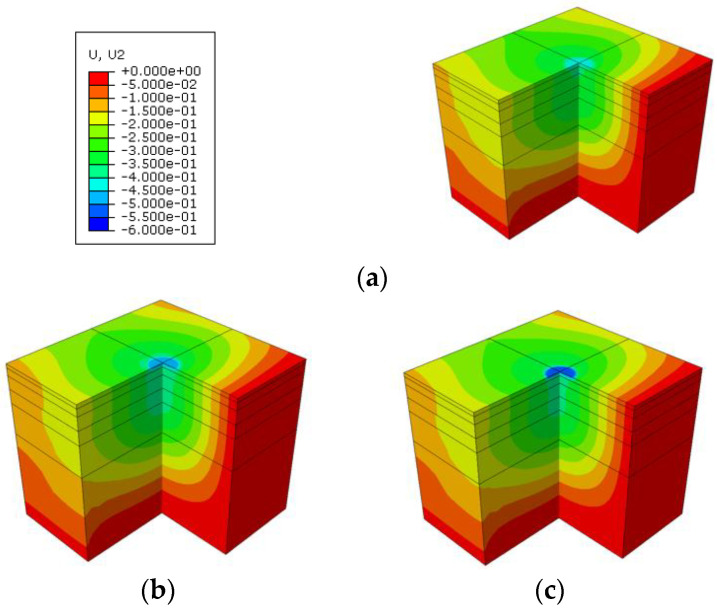
Distribution of the vertical displacement at different moments: (**a**) 6 h, 24.7 °C; (**b**) 22 h, 30.2 °C; and (**c**) 13 h, 50.1 °C.

For the heat transfer simulation in summer temperatures, the pavement surface temperature at 24.7 °C and 50.1 °C corresponded to 6 h and 13 h, respectively. Since the temperature distribution between the asphalt layers at night is different from that at daytime, 22 h was selected as the research object again, and the surface temperature of the asphalt pavement was 30.2 °C. The vertical displacement distribution of the road at the different temperatures and different times is shown in Figure 7.

**Figure 8:**
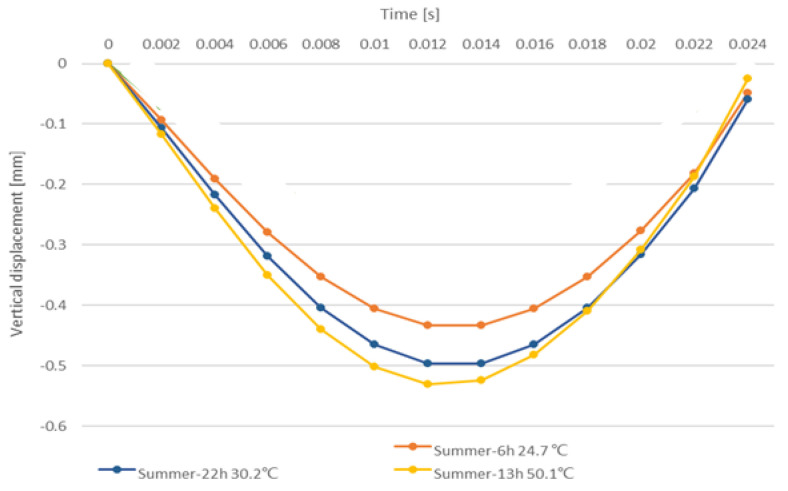
Vertical displacement of the center point of the asphalt surface course at different moments.

It can be seen from Figure 7 that under the influence of different temperatures, the vertical displacement of the pavement under the same load is obviously different. Figure 8 shows the time-dependent variation of the vertical displacement of the center point in the loading area of the pavement surface. The vertical displacement increases with increasing load at the beginning and reaches a peak at 0.012 s. Immediately afterward, the vertical displacement again gradually decreases to 0 as the load is gradually removed. The change of vertical displacement with time is consistent with the change of the applied load. It can be seen that although the surface temperature difference between 6 h (24.7 °C) and 22 h (30.2 °C) is less than 6 °C, the difference in the vertical displacement is much larger than that between 22 h (30.2 °C) and 13 h (50.1 °C). The main reason is that the temperature distribution along the pavement depth is different in the morning than it is at night. At night, the maximum temperature of the road structure appears in the bottom of the asphalt surface course, while the maximum temperature of the daytime structure always appears on the surface of the pavement, which can be seen in Figure 5. This difference in temperature distribution leads to changes in structure stiffness, resulting in significant differences in vertical displacement. It is worth mentioning that the computational mechanical responses were derived from the pavement structures (conventional pavement and cool pavement), with full connection between the asphalt layers. For the influence of the different interlayer bonding conditions on the mechanical responses of the asphalt pavement, many previous studies from both experimental and numerical aspects have been carried out, and it is not the research focus of this study.

### 4.2. Results of the Responses of Cool Pavement 

#### 4.2.1. Results of the Thermal Response of Cool Pavement 

**Figure 9:**
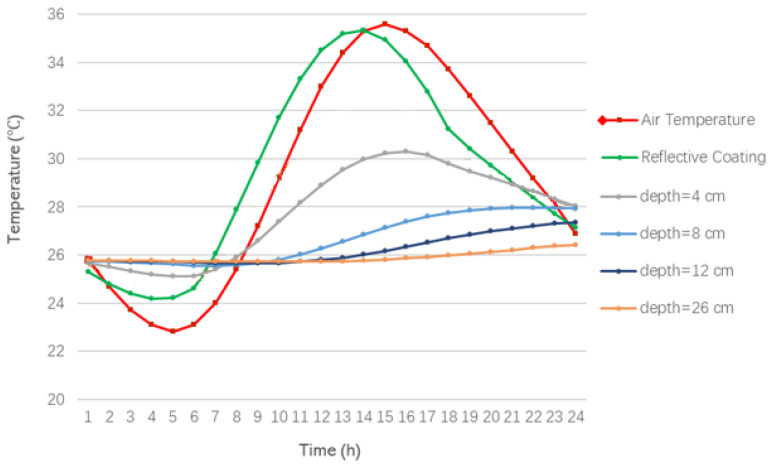
Hourly air temperature and temperature of the asphalt layers of the cool pavement at different depths in the summer.

Figure 9 shows the temperature trend of the cool pavement at different depths over time. The temperature trend of the cool pavement at different depths is approximately the same as that of the conventional pavement under the same conditions. The cool pavement has a significant cooling effect such that the temperature of the asphalt pavement surface is approximately the same as the air temperature. 

**Figure 10:**
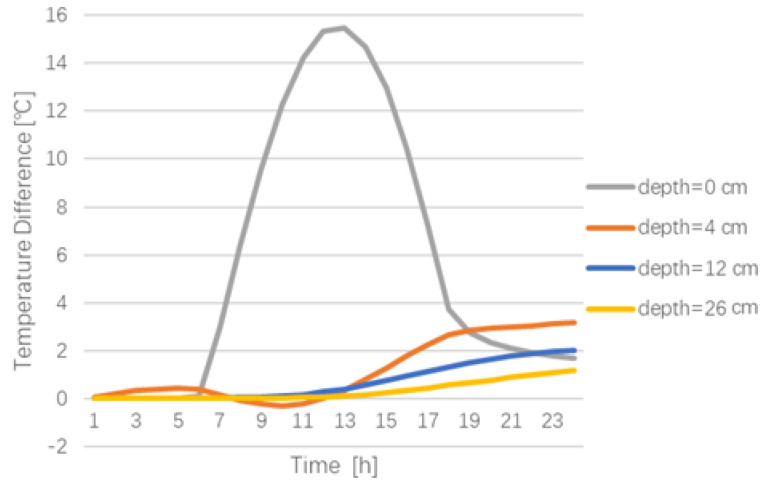
Temperature difference between the conventional pavement and cool pavement at different moments.

The temperature difference between the conventional pavement and cool pavement at different depths is shown in Figure 10.

As can be seen from Figure 10, the cool pavement coating mainly decreases the temperature at the top of the asphalt pavement (y = 0 cm), which can be cooled by up to 15.5 °C. With the increase in pavement depth, the temperature reduction become less. In the range of 12–26 cm in the asphalt base course, the cooling effect brought by the cool pavement is not obvious in the daytime, but it can bring about a 2 °C cooling effect at night.

#### 4.2.2. Results of the Mechanical Response of Cool Pavement

According to the above research results, the use of a cool pavement coating can effectively reduce the temperature of the pavement surface. At the highest temperature at 13 h in the summer, the pavement temperature can be reduced by as much as 15.5 °C, and the cooling effect is the best at this time. Therefore, the influence of the mechanical parameters caused by the application of cool pavement at this time was studied.

**Figure 11:**
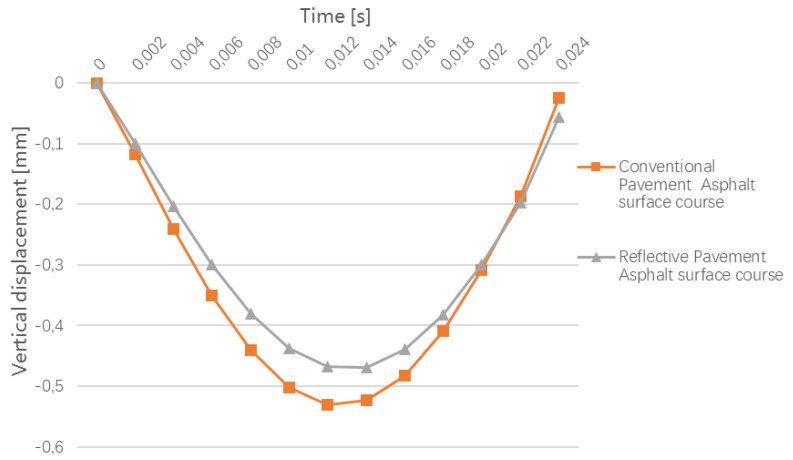
Comparison of the vertical displacement of the conventional pavement and cool pavement at 13 h in the summer.

The comparison of the vertical displacement of the conventional pavement and the cool pavement at 13 h in the summer is shown in Figure 11.

As shown in Figure 11, on the asphalt surface course, the cooling effect is obvious, and the change of vertical displacement is brought by the temperature decrease. At the center of the load, the vertical displacement of the road surface is decreased by 0.06 mm (11.3% of the displacement in conventional pavement), which reduces the permanent deformation (rutting) of the asphalt pavement under the cumulative load over time.

**Figure 12:**
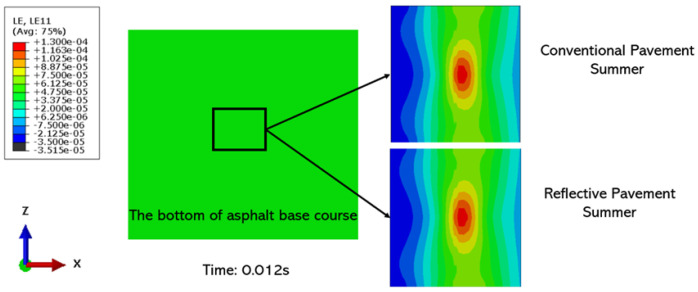
Distribution of logarithmic horizontal strain of the conventional and cool pavements.

Figure 12 shows the horizontal strains for the conventional and cool pavements in the summer under a uniform contact pressure. The application of the cool pavement does not affect the horizontal strain and there is no difference between the maximum and minimum values. The likelihood of fatigue damage on the asphalt base course is approximately the same for both pavements. However, the cool pavement has a better resistance to rutting because there is still a large difference in the road surface temperature.

## 5. Conclusions and Outlook

In this study, the 3D models of asphalt pavements were established through the FE method. The stable temperature field of the pavement structure was calculated by applying different temperature boundary conditions to the pavement surface and applying the mechanical model to analyze the mechanical response of conventional pavement and cool pavement in a real environment. The simulation of the pavement structure temperature distribution under summer weather conditions shows that the pavement surface temperature can exceed 50 °C, which can easily lead to permanent deformation of the pavement surface and affect the service life of asphalt pavement. The cool pavement has a significant cooling effect, which makes the surface temperature of asphalt pavement and the atmospheric temperature nearly flat, and the temperature of each depth has a certain degree of reduction. The cool pavement mainly reduces the temperature of the top of the asphalt pavement. Particularly, cool pavement reduces the surface temperature of pavement by more than 15 °C, and it reduces the vertical displacement of pavement by approximately 11.3%, which proves that cool pavement can effectively improve anti-rutting performance. However, the use of cool pavement does not change the horizontal strain at the bottom of the asphalt base course, and so it does not increase the fatigue resistance of asphalt pavement.

In summary, the mechanical response of asphalt pavement was studied considering the characteristics of asphalt material changing with temperature. In future research, more environmental conditions should be considered, such as variable wind speed and humidity. The different interlayer bonding conditions between the asphalt layers should be investigated by experimental tests and numerical simulations. Additionally, wheel load should be calculated by establishing a vehicle dynamics model.

## Figures and Tables

**Table 1:** Thermal properties of the cool pavement on a global scale.

Albedo	19%
Pavement radiation emissivity	0.828
Thermal conductivity	907.2 mJ/(mm∙h∙K)

**Table 2:** Representative summer weather data used in the FE model [20].

Wind Speed	Sunshine Time	Air Temperature			
2.6 m/s	10.7 h	Time	°C	Time	°C
		1:00	25.8	13:00	34.4
		2:00	24.7	14:00	35.3
		3:00	23.7	15:00	35.6
		4:00	23.1	16:00	35.3
		5:00	22.8	17:00	34.7
		6:00	23.1	18:00	33.7
		7:00	24	19:00	32.6
		8:00	25.4	20:00	31.5
		9:00	27.2	21:00	30.3
		10:00	29.2	22:00	29.2
		11:00	31.2	23:00	28.1
		12:00	33	0:00	26.9

**Table 3:** Thickness and material properties of the pavement structure.

Layer	Poisson’s Ratio	Density	Damping Factor
		[ton/mm^3^]	
Asphalt surface course	0.35	2.38 × 10^−9^	0.9
Asphalt binder course	0.35	2.49 × 10^−9^	0.9
Asphalt base course	0.35	2.30 × 10^−9^	0.9
Hydraulically bound base course	0.25	2.40 × 10^−9^	0.8
Frost protection course	0.5	2.40 × 10^−9^	0.4
Subgrade	0.5	2.40 × 10^−9^	0.4

**Table 4:** Prony series data of the asphalt surface course.

Item	Relaxation Time (s)	The mth Maxwell Spring Modulus (MPa)	Item	Relaxation Time (s)	The mth Maxwell Spring Modulus (MPa)
1	1.00 × 10^−7^	1.25	7	0.1	1141.19
2	1.00 × 10^−6^	3.90	8	1	2214.31
3	1.0 × 10^−5^	12.29	9	10	1624.26
4	1.0 × 10^−4^	38.33	10	100	425.02
5	0.001	121.51	11	1000	39.42
6	0.01	371.69	12	10,000	1.00
The parallel spring modulus (MPa)				107.04	
Tr (°C)				−5	
C1				10.16	
C2				46.64	

**Table 5:** Prony series data of the asphalt base course.

Item	Relaxation Time (s)	The mth Maxwell Spring Modulus (MPa)	Item	Relaxation Time (s)	The mth Maxwell Spring Modulus (MPa)
1	1.00 × 10^−7^	1.73	7	0.1	13,237.69
2	1.00 × 10^−6^	9.67	8	1	7135.05
3	1.0 × 10^−5^	42.93	9	10	2066.84
4	1.0 × 10^−4^	248.43	10	100	678.16
5	0.001	1148.68	11	1000	187.77
6	0.01	6775.57	12	10,000	65.36
The parallel spring modulus (MPa)				22.29	
Tr (°C)				10	
C1				254,154,208	
C2				1,637,000,000	

## Data Availability

The data are available from the first author and can be shared upon reasonable request.
